# The PIER framework for healthcare simulation integration in undergraduate physiotherapy education

**DOI:** 10.1186/s12909-022-03751-7

**Published:** 2022-09-24

**Authors:** Anke van der Merwe, Roline Yvette Barnes, Mathys Jacobus Labuschagne

**Affiliations:** 1 Department of Physiotherapy, Faculty of Health Sciences, University of the Free State, 205 Nelson Mandela Drive, Park West, Bloemfontein, 9301 Free State South Africa; 2 Clinical Simulation and Skills Unit, Faculty of Health Sciences, University of the Free State, Bloemfontein, Free State South Africa

**Keywords:** Healthcare simulation, Curriculum, PIER framework, Physiotherapy

## Abstract

**Background:**

The need for healthcare curricula renewal to facilitate a continuum in education from classrooms to diverse healthcare settings is undeniable. Simulation has been recognized as an educational strategy to address healthcare education challenges, with limited reporting on the integration of simulation-based learning experiences in physiotherapy education. The study aimed to describe the finalisation of a framework for integration of healthcare simulation in an undergraduate physiotherapy program.

**Methods:**

A qualitative descriptive research design was utilized. Five South African experts in the fields of healthcare simulation and/or physiotherapy education contributed to the finalization of the framework during a consensus meeting. Content analysis was employed and credibility was ensured through double coding.

**Results:**

Structural coding yielded five themes- Planning, Implementation, Program Evaluation, Program Revision and Framework. The five themes consisted of fifteen categories, two sub-categories and 44 codes. The planning theme was most robust with seven categories. The Planning, Implementation, Evaluation, Revision (PIER) framework was developed and finalized by expert participants. following the consensus meeting.

**Conclusion:**

Needs analyses when planning and incorporating simulation is essential. Collaboration through resource and knowledge sharing is vital in developing a responsive curriculum integrating simulation. Furthermore, facilitator and student preparation are paramount in ensuring active engagement in simulated-based learning experiences. The interconnectedness of all framework elements and integration phases, as well as the implied importance of competent facilitators and prepared students is crucial and highlights careful consideration to be given to these aspects. The PIER framework is generic in nature and represents the continuous process of simulation integration for any healthcare program.

**Supplementary Information:**

The online version contains supplementary material available at 10.1186/s12909-022-03751-7.

## Background

Healthcare education is challenged with fewer clinical learning opportunities due to ever changing burden of diseases, limited funding, and previously identified lack of students’ theoretical knowledge transfer to clinical practice [1–4]. The substantial burden on healthcare systems as a result of the COVID-19 pandemic [5], led to a radical change in healthcare training. Healthcare educators face additional challenges which include, but are not limited to, an underprepared student population, lack of adequate infrastructure and resources, severe financial constraints, a focus on primary healthcare and an ethnically and culturally diverse student population [6, 7]. These challenges necessitate that traditional educational methodologies be revisited and that alternative methods, such as simulation and blended online approaches, be explored and integrated. 

Aligning with the experiential and constructivist learning theories, a highly contextualised learning environment could be effective in promoting learning [8]. Subsequently evidence has been presented that simulation could facilitate the transfer of knowledge to the clinical setting [3, 9, 10], resulting in many healthcare programmes worldwide integrating simulation in their undergraduate programmes. Simulation-based education (SBE) is rich in diversity with educators drawing from numerous learning theories and educational principles to inform their instruction by means of simulation [11]. Simulation also has the ability to transform learning to an interactive and realistic process, providing “hands-on” student-centred education in a more realistic environment [1, 12].

The benefits of integrating simulation in healthcare education is undeniable with international simulation-based research advocating that Simulation-Based Learning Experience (SBLEs) may overcome the identified healthcare and educational challenges [3, 4, 8, 11]. Healthcare curricula have incorporated aspects of simulation over the past 40 years, but the full curricular integration of this valuable educational methodology is still met with resistance [10, 13]. Published best practice guidelines guide the design of SBLEs with mention only being made of the importance of curricular integration [14]. As with any educational methodology, acceptance and full integration of the methodology is essential to ensure success and sustainability [10]. 

The authors developed a conceptual framework for simulation integration, described in previous publications [15, 16]. However, program responsiveness to the changing context of higher education is vital and contextualizing research is therefore essential to ensure the needs of the population are met [17]. Although both the systematic review and Delphi survey [15, 16] included primarily international input, disparity in the available resources, varied educator competency and cultural differences [18] challenges the implementation of simulation-based frameworks designed for developed countries within a developing economy. Following review of the conceptual framework, the authors identified a need for an additional phase to finalise the framework in order to contextualise the framework content. We aimed to explore the opinions of experts, in the fields of healthcare simulation and/ or physiotherapy education, to describe the finalisation process of a framework for the integration of simulation in an undergraduate physiotherapy program within a developing economy. 

## Methods

This qualitative descriptive research study encompassed the final phase of a larger study following the development of a conceptual framework by means of a systematic review and previously described Delphi survey [15, 16] to identify elements to be included in a conceptual framework guiding simulation integration. This study was situated in the constructivist research paradigm. Ethical approval was obtained from the Health Sciences Research Ethics Committee at the University of the Free State prior to study commencement (HSREC 108/2017), ensuring that all guidelines for research including human participants were adhered to. A consensus meeting, utilizing a semi-structured group interview process, enabled collaborative construction and meaning negotiation of the presented framework [19]. Through purposive convenience sampling five South African experts from various academic institutions in South Africa were identified to ascertain the necessary information by means of questioning [20, 21]. Participants with healthcare research experience and publications, healthcare or physiotherapy simulation experts; and members of national educator forums were approached to provide insight into the current focus in healthcare education. The researcher identified five suitable participants, each with more than 15 years’ experience in their respective healthcare research fields (Table 1).

**Table 1:** Experts included

Profession	Areas of expertise
Medicine	Dean of Medical Faculty; educationalist with extensive publications; simulation expert; member of the Council of the Academy of Science of South Africa
Nursing	Educationalist with extensive publications; simulation expert
Nursing	Educationalist with extensive publications; simulation expert
Physiotherapy	Educationalist with extensive publications; member of Health Professions Council of South Africa education board
Physiotherapy	Educationalist with extensive publications; National Physiotherapy Educators Forum member

The consensus meeting was facilitated by an independent facilitator, and was structured according to a self-developed meeting guide comprising of open-ended questions [20]. Participants were provided with the conceptual framework and elements informing the framework [15, 16]. The use of the meeting guide, conceptual framework and informing framework elements ensured that all interview areas were covered, satisfying both the thematic and dynamic study dimensions [20]. Two independent exploratory discussions were conducted by the first author with two experienced qualitative researchers prior to the consensus meeting to ensure the reliability of the meeting guide and meeting procedure.

The consensus meeting was held in a private room ensuring confidentiality and a neutral interview space. Informed consent, including the use of audio-recordings, was obtained from participants prior to the commencement of the meeting. To further ensure confidentiality during transcription, participants received a number to identify themselves with during the meeting. A convergent process was utilized to obtain the best possible answers regarding the framework content and informing elements presented for discussion [22, 23]. Consensus was achieved when all participants agreed on the value of including or excluding an element. Where no consensus could be reached, the discussion was facilitated until data saturation was achieved. Data saturation was defined as no new comments, recommendations or opinions being raised by participants regarding the value of a framework element [23]. The facilitator did not at any time reign in the discussion, as the freedom provided data with depth and richness [24]. The first author was present during the meeting as an observer and made field notes of participant’s reactions and suggestions, which in combination with the audio-recordings and subsequent transcriptions, allowed for a dependability and confirmability audit [21]. A visual framework representation was available on a white board for reference and discussion purposes during the meeting, allowing for knowledge co-construction [19]. Once consensus was reached on the inclusion or exclusion of an element or the name assigned to an element or framework theme, the visual framework was adjusted accordingly. The consensus seeking process and inclusion of the framework finalization phase through a member checking process ensured accurate interpretation, and therefore credibility, of the constructed data [21, 25].

**Fig. 1:**
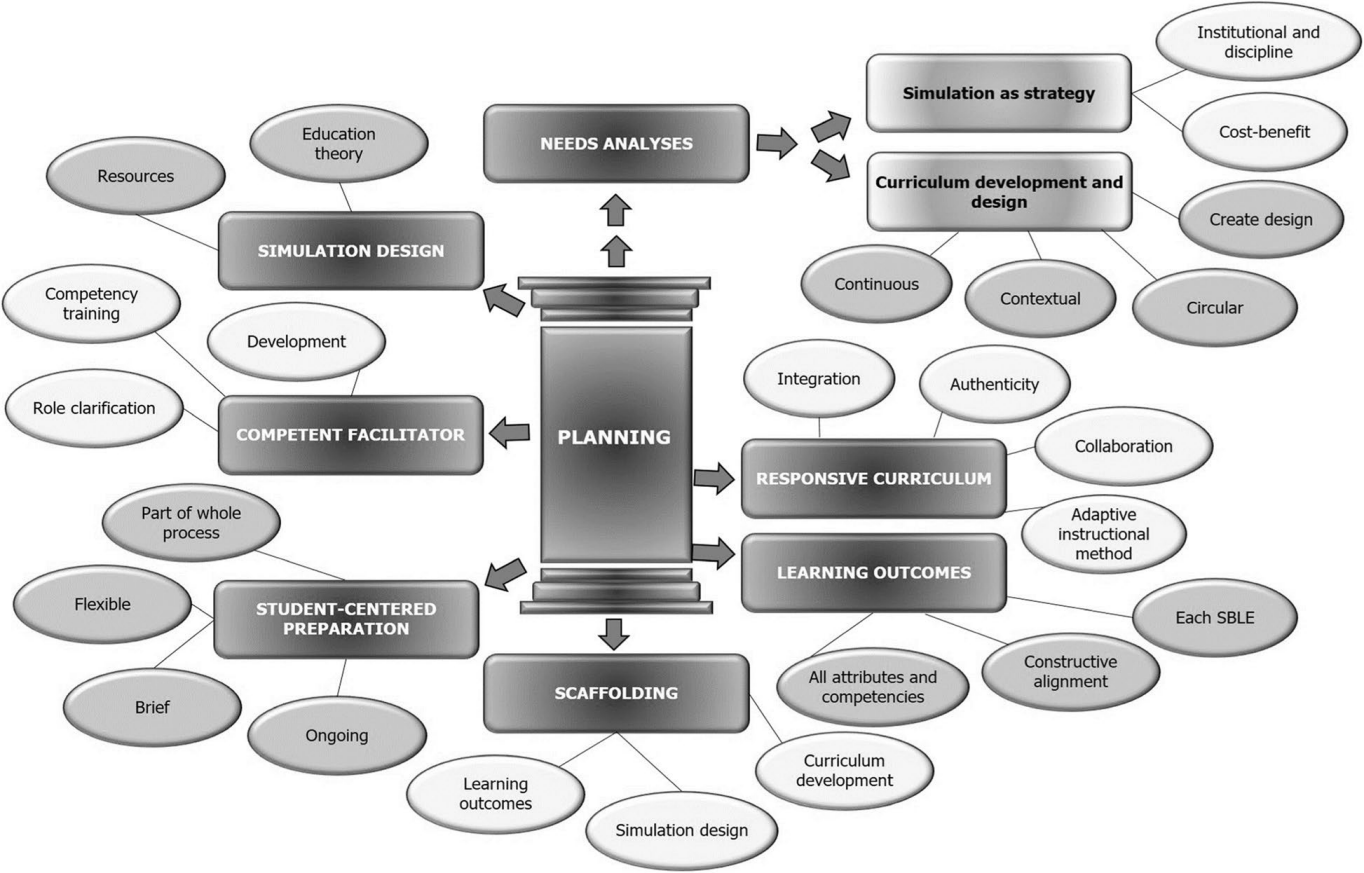
Content analysis results- Planning

The process of structural coding, as described by Saldaña (2010), was utilized to analyze the qualitative data collected during the consensus meeting. The process of structural coding commenced with data verification after which the data was independently reviewed by both the author and a qualitative data analyst, and structural codes were identified through identification of specific structural attributes in the data. Similar structural codes were grouped together into categories and sub-categories based on the semi-structured meeting guide [25]. Related categories were further grouped in the overarching themes previously identified in the presented conceptual framework (Fig. 1 and Table 2). Following the independently performed structural coding, a consensus meeting with the qualitative data analyst established inter-coder agreement, enhancing study confirmability and credibility [21]. Consensus, defined as 100% agreement, was achieved regarding the identified categories, sub-categories and codes ensuring unbiased and accurate data.

**Table 2:** Content analysis results- Implementation, Program evaluation, Program revision, Framework

Theme	Category	Sub-category	Code
**Implementation**	Debriefing	None	Self-reflection
			Constructive feedback
			Self-regulation
			Goal setting
	Assessment	None	Suitability for model?
			Tool
			Terms and type
			Peer-assessment
**Programme evaluation**	Validation	None	Essential
			Evidence-based
	Evaluate	None	Feedback
			Essential
**Programme revision**	Review	None	Continuous
**Framework**	Practicality	None	Cost
			Decentralised training
	Impact		Institutional support
			Collaboration
	Supplementation		Interprofessional use
			No replacement/ substitution

## Results

A total of five themes were identified during the content analysis process, consisting of fifteen categories, two sub-categories and 44 codes (Fig. 1 and Table 2). Study results assisted in the adjustment and refinement of the conceptual framework [15, 16] and are presented and discussed as such [see Additional file 1].

The Planning theme was the most robust, with a total of seven categories identified (Fig. 1). The category of *Needs analyses* was the only one to include sub-categories. Two category name changes were required to better reflect standard healthcare education terminology. Curriculum development and outcomes were changed to *responsive curriculum* and *learning outcomes*, reflecting a more adaptable curriculum with specific educational outcomes [15].

Student preparation was adjusted to *student-centred preparation* and moved to the Planning theme, demonstrating the student-centeredness of the educational methodology [15, 16]. The category *competent facilitator* was created by merging the previously identified categories of *training* and *educator role* [15]. The previously identified categories, instructional method and resources, were collapsed within the *responsive curriculum* and *simulation design* categories respectively [16].

The categories of feedback, initially included in the Planning theme, and student goal setting, were collapsed within the *debriefing category* in the *implementation theme* [15, 16]. Mastery learning/Deliberate practice (ML/DP) was also collapsed within the *debriefing category*, due to ML/DP requiring constant self-regulation, self-reflection and goal setting [15, 16]. The suitability of including assessment within the framework was questioned by participants (Table 2) [15, 16]. *Program evaluation and revision* were identified as two separate themes with *validation*, *evaluation* and *review* as respective categories (Table 2). Within the *Framework* theme the practicality and impact of the framework emerged and revealed the collaborative and interprofessional nature of the presented framework, with the use of SBLEs to supplement clinical hours not supported by participants (Table 2).

**Fig. 2:**
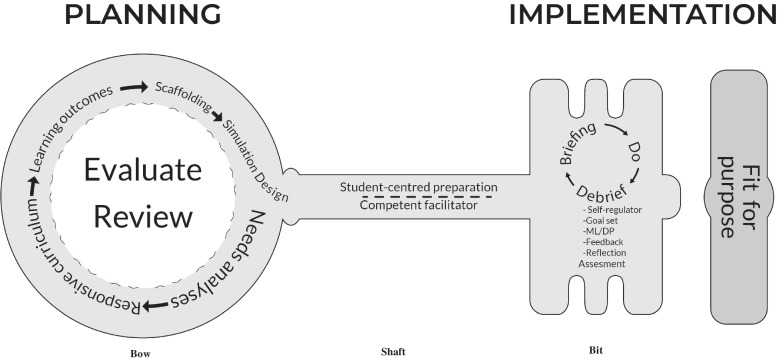
PIER framework for healthcare simulation integration in undergraduate physiotherapy education

The PIER framework for simulation integration (Fig. 2) was finalised through in-depth exploration of participant opinion and included two independent member-checking processes.

## Discussion

The data obtained during the consensus meeting was used to finalise the framework for the integration of simulation in an undergraduate physiotherapy program within a developing economy. Engaging with South African experts to amend and refine the conceptual framework [see Additional file 1] allowed for expert input to develop a more generic, acceptable and sustainable end-product for simulation integration. Due to the significance of the framework shape, this section will present and discuss the end-product according to the framework shape.

### Framework

The unidirectional shape and design of the conceptual framework were criticised by participants. The fact that some elements were present in more than one integration phase led participants to discuss the iterative nature of the framework. A key shape was proposed and accepted by all participants, as the framework should.

*“be the key for the way one looks at things” *[2]

when adjusting healthcare programmes to integrate simulation.

The interconnectedness of all framework elements and integration phases are depicted by using dashed lines illustrating a degree of fluidity between elements and phases [26]. The importance of competent facilitators and prepared students is visually depicted in the framework by its central position linking the planning and implementation phases and highlights careful consideration to be given to these aspects (Fig. 2). Actively engaging with curriculum content through SBLE participation and debriefing allows students to reflect and construct new knowledge for implementation in future SBLEs and clinical settings [27].

### Bow of the key

When viewing the visual representation of the framework, the bow of the key (Fig. 2) represents the planning phase, where simulation integration should commence. It is generally accepted that a reader starts reading at the upper left of an illustration [26], however, as the simulation design element is intended to flow directly into the segment containing the elements of student-centred preparation and competent facilitator, the authors were required to consider alternative visual representation. Enlarged font was selected to increase the focus on and to guide the framework user to the point where the planning phase starts [26]. Arrows were deemed necessary to direct the cause-effect relationship of the elements of the planning phase of integrating simulation [26]. Due to constant framework evolution, the planning phase was consolidated and collapsed during the final research phase, to be presented as a concise framework section.

The needs analysis element serves as the starting point for simulation integration. With tertiary training institutions playing an integral role in ensuring economic growth and social justice – with consideration of social, racial and gender equality – in the quest for innovation and growth. Identifying the needs of all stakeholders are essential for institutional sustainability. The World Bank (2021) explicitly indicated their commitment to improving the quality and relevance of higher education programmes to increase employability of graduates. The unresponsiveness of healthcare curricula regarding student needs and societal circumstances has been highlighted [17, 28, 29] and even cited as one of the main reasons for the violent student protests calling for curricular decolonisation in South Africa [17, 28]. The development of a responsive curriculum emerges from the acceptance of and ability to integrate simulation into the existing curriculum, in response to identified societal and student needs, to ensure curriculum relevance to the context where the curriculum will be enacted. By nature, a responsive curriculum is authentic, in not only answering the identified curricular needs, but also in the choice and design of individual SBLEs.

“*I think the authenticity must underlie the whole [curriculum development] process and then the specific event has got to be authentic.*” [2]

Detailed learning outcomes will ensure curriculum transparency and provide both educators and students with guidance regarding what is expected if students are to be deemed successful in the programme [10, 14]. Identifying how and where simulation will be implemented to best address the learning outcomes, in accordance with the students’ experience level, would subsequently be incorporated in the simulation design phase. The framework (Fig. 2) describes the integration of simulation into an already existing programme, and does not propose a purely simulation-based programme, as not all learning outcomes are expected to be met with the use of SBLEs.

Even though scaffolding is conceptually part of the curriculum [14], it was deemed essential that it was visible in the framework. Designing SBLEs according to not only the theoretical, but also the psychological level of the student, is essential for optimising learning and to ensure a safe learning environment, in which students are aware of exactly what is expected of them and how they can achieve the learning outcomes [24, 30].

Simulation design was depicted as flowing into the section containing the elements of student-centred preparation and competent facilitator (Fig. 2). A dashed line is used to depict that neither of these elements are concrete, and to imply change [26], as facilitator and student roles change throughout the integration of the framework. SBLE designs are equally dependent on student and facilitator preparation, adjustments required according to changing needs, as well as stakeholder feedback obtained. All aspects relating to individual SBLE design were removed from the visual framework representation, to declutter the framework.

The section containing the elements of evaluate and review may be viewed as a separate circle, situated within the bow of the key (Fig. 2). A dashed line is used to distinguish between the planning, programme evaluation and programme revision phases. Even though evaluation and review are situated in the planning phase of the framework, deficits identified during the simulation implementation phase will, by implication, result in framework facilitators returning to the planning phase to revise strategies. Continuous evaluation and review will ensure the constructive alignment of SBLEs founded in sound education theory and simulation best practice [14]. The use of dashed lines, implying that something has not yet occurred [26], depicts that both the evaluation and review processes may be implemented, throughout all framework sections, as required.

### Shaft of the key

Although the elements of student-centred preparation and competent facilitator conceptually form part of the planning phase, participants emphasised their importance for the entire framework, visually portrayed by these two elements connecting directly and moving through the planning and implementation phases (Fig. 2). Facilitators who are not competent in delivering the educational methodology are likely to revert to education strategies and roles that they are more familiar with. Overloading the SBLE to address numerous outcomes with the goal of saving programme time may also be the result of facilitator incompetency in optimal SBLE design [31]. Ensuring the competence of curriculum and SBLE designers, including the facilitators involved in SBLE facilitation and debriefing, will limit curriculum drift, as the programme that is designed will be enacted by capable facilitators who can execute it.

The preparation required of students, and the roles of the facilitators involved, will continuously change throughout the implementation of the framework, and is, therefore, illustrated as being able to move to and from all aspects of the framework. The Healthcare Simulation Dictionary provides two descriptions of a facilitator, namely, an individual participating in any part of simulation implementation and/or delivery, or an individual directly facilitating the achievement of a desired outcome in an SBLE [32]. Due to the various roles fulfilled by educators in the simulated environment [33, 34], careful consideration was given to labelling the element of competent facilitator. Opting for the term, facilitator, implies that the educator is not necessarily required to participate in all framework phases. Notably in educational environments where there is a shortage of healthcare educators [6], especially educators trained in the use of simulation as educational methodology [33], and limited institutional financial resources [6, 28], makes identification of facilitative roles when integrating the framework essential. The authors suggest that appropriate educators be identified for the various facilitative roles required by the framework.

Dedicated time for student preparation, both theoretically and psychologically [30, 35, 36], prior to engaging with the simulated environment, is a necessity as the current tertiary education student population yearns for frequent guidance and clear and transparent direction [37, 38]. The authors, furthermore, advise that students are trained to ensure that they can provide detailed and constructive feedback. Student feedback after participation in the programme that integrates simulation is another vital student role that will assist with programme evaluation.

### Bit of the key

The bit of the key represents the active participatory phase. Specific mention is made of student briefing prior to SBLE participation (Do), to orientate students to the SBLE’s expectations, and the simulated environment they will encounter, and to situate the SBLE within the programme outcomes (Fig. 2). The role of briefing differs from the previously described student-centred preparation, in that it specifically relates to individual SBLE orientation and role clarification. Diverse student populations might not be accustomed to the simulated learning environment and may require focused psychological preparation and attention to their role during the SBLE, if they are to participate in the learning experience optimally [33].

The importance of the sequential briefing, doing and debriefing cycle is depicted by solid line arrows [26], that visually present the causal influence the elements have on each other. The element of debriefing is further elaborated on, in the form of a list below the word, to indicate the components comprising debriefing (Fig. 2). The goal of the list is purely to indicate which essential components form part of and should be incorporated in the debriefing process, and elements are not given in any order. To foster the development of lifelong learners with critical reasoning abilities, engagement in reflection activities after SBLE participation is not only limited to certain types of SBLEs but is essential for students after all simulated activities [39]. When students are facilitated to reflect on their actions by participating in debriefing led by a trained facilitator, and if they receive constructive feedback and engage in ML/DP, students may develop self-regulatory skills, particularly for identifying individual strengths and shortcomings.

Because the framework is situated within the constructivist paradigm and relies heavily on Kolb’s experiential learning theory [27], the inclusion of an opportunity for repetition is essential. To decrease the resource burden, essential skills to be mastered by means of ML/DP should be identified, and self-directed skills practice, including a component of peer assessment and self-reflection, is advised. Adding a peer assessment component may be beneficial as it has been shown to increase student learning, contribute to collaboration skills and foster reflection [40].

One participant mentioned that *“constructive feedback is embedded within debriefing” *[5] and another highlighted that *“with constructive feedback we’re already being encouraged to get students to think about what they did, to become reflective practitioners” *[4], which speaks to the primary goal of the debriefing process. Therefore, ML/DP and constructive feedback are positioned within the overarching debriefing element, as it provides constructive feedback and aims to facilitate student reflection, to affect future practice.

Assessment is positioned at the end of the implementation phase, and refers, specifically, to summative practical-skills-based assessment and peer assessment. To protect the safety of the simulated learning environment and manage the psychological safety and anxiety of students [14], students should be afforded the opportunity to engage in similar peer-assessment opportunities and practical-skills-based SBLEs prior to their assessment. As there is no evidence available indicating the current use of simulation in the South African undergraduate physiotherapy programmes, the authors deem it inappropriate to assess students in a summative manner in the immersive simulated environment.

In line with Kolb’s experiential learning theory, the core of ML/DP is the facilitation of self-reflection and development of self-regulation [41], and a peer-assessment component is encouraged for both immersive and practical-skills-based SBLEs, as it actively engages all SBLE participants. Healthcare education increasingly places emphasis on the attainment of professional behaviours and attributes additional to technical skills. Educators should consider that not all skills are required to be mastered, especially when considering immersive simulation experiences, “*there’s more than one way to do things” *[4] and forcing mastery to a set benchmark in such an environment could result in an “*increased risk for perceiving it as negative experience” *[4].

### Keyhole

A keyhole was added to the visual framework presentation to depict the contextual validation of the framework. The deeper the key is inserted in the lock, the further the integration process has progressed, with success being demonstrated by the lock opening.

Framework amendments brought about following this study phase with healthcare education experts resulted in a more generic framework taking into account resource restrictions and accepted simulation education practice ensuring a framework which may be applicable to any healthcare profession.

The implementation of the PIER framework, with subsequent evaluation of the effect and acceptability thereof, is recommended for future research.

## Conclusion

The need for amending healthcare curricula and programmes to facilitate a continuum in education from the classroom to the diverse healthcare setting is undeniable. Investigation into institutional ability for simulation integration is an essential first step in simulation integration. Notably, within resource restricted environments, it is of the utmost importance to provide stakeholders and funders with proof of the value of simulation to ensure sustainability. The PIER framework emphasises the preparation required by both educators and students and the importance of addressing the needs of all involved stakeholders. Curriculum and SBLE authenticity are essential framework components for optimising preparation of graduates for practice, with the expectation that graduates should possess increased and complex skills early in their careers. The PIER framework may be applicable for use in any healthcare programme due to the generic nature of the framework.

The key shaped PIER framework assists framework facilitators to change the way education is viewed, by finding the right key, to develop graduates who are able to answer to the needs of society whilst continuously reflecting and engaging in lifelong learning.

## Supplementary Information

**Additional file 1:** **Supplementary material 1.** Conceptual framework for the integration of simulation in the South African undergraduate physiotherapy programme.

## Data Availability

Additional datasets used and analysed during the current study are available from the corresponding author on reasonable request.

## Abbreviations

SBLE: Simulation-based learning experience
ML/DP: Mastery learning/deliberate practice
SBE: Simulation-based education
