# Supplementary material for: The PIER framework for healthcare simulation integration in undergraduate physiotherapy education
Source: BMC Med Educ. 2022 Sep 24;22:690. doi: 10.1186/s12909-022-03751-7 (PMC9509622; doi:10.1186/s12909-022-03751-7)
Supplement: Supplementary file 1 — Additional file 1: Supplementary material 1. Conceptual framework for the integration of simulation in the South African undergraduate physiotherapy programme. [file 12909_2022_3751_MOESM1_ESM.docx]

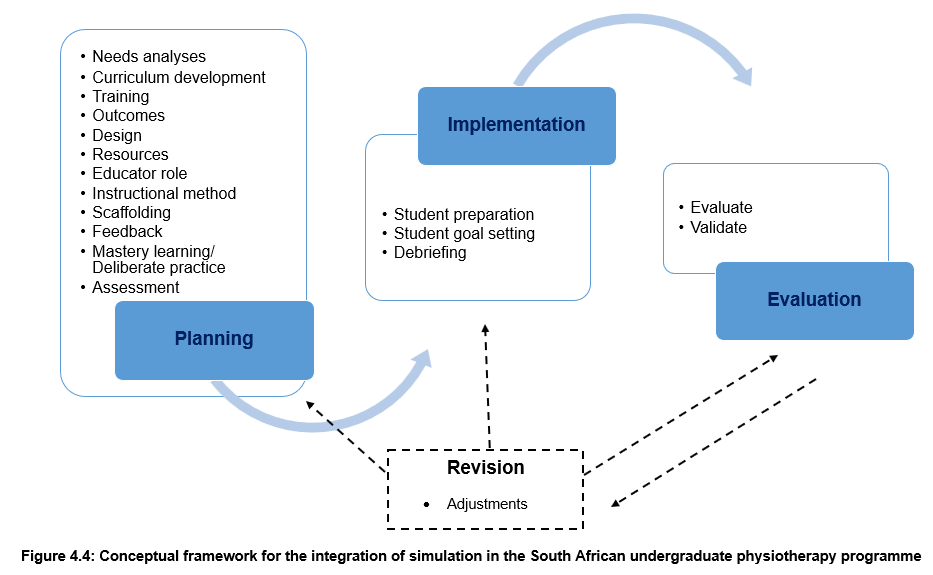


Supplementary material 1. Conceptual framework for the integration of simulation in the South African undergraduate physiotherapy programme

**(compiled by the researcher, van der Merwe, 2020)**
